# Death of President Harrison

**Published:** 1841-05

**Authors:** 


### DEATH OF PRESIDENT HARRISON.

We have seen the official report of the physicians who attended the President of the United States in his last illness. The disease of which this venerable patriot died, was acute pneumonia, produced, as the gentlemen suppose, by his exposure to a shower, in a morning walk. We rather think, that the oceanic March winds of his new locality, so unlike those to which he had been accustomed, on the dry and sunny slopes of the Ohio, were to blame for a part of the mischief. The respectable medical gentlemen who attended him, thought him too weak to bear the lancet, and therefore did not employ it—a decision which their brethren at a distance are not perhaps at liberty to question. No *post mortem* inspection was made.

The President always felt himself in sympathy with the Medical Profession, to which he was originally devoted, and in the study of which he was engaged at the time of his appointment in the army, in 1791. Thirty years afterwards, when a member of the Legislature of Ohio, he rendered important aid to the writer of this article, in obtaining a charter and endowment, for a public hospital in Cincinnati. D.

